# Emerging Filamentous Basidiomycetes as Opportunistic Human Pathogens: Diagnostic and Antifungal Challenges in a Tertiary-Care Center in Thailand

**DOI:** 10.3390/antibiotics15050424

**Published:** 2026-04-23

**Authors:** Chaianant Leelabooranasak, Arsa Thammahong, Kornvalee Meesilapavikkai, Jakapat Vanichanan

**Affiliations:** 1 Division of Infectious Diseases, Department of Medicine, Faculty of Medicine, Chulalongkorn University and King Chulalongkorn Memorial Hospital, Bangkok 10330, Thailand; chaianant.mdcu@gmail.com; 2 Center of Excellence in Antimicrobial Resistance and Stewardship, Department of Microbiology, Faculty of Medicine, Chulalongkorn University, Bangkok 10330, Thailand; arsa.t@chula.ac.th

**Keywords:** filamentous basidiomycetes, antifungal resistance, molecular identification

## Abstract

**Background/Objectives**: Filamentous basidiomycetes are environmental fungi that rarely cause human infection but are increasingly recognized as opportunistic pathogens, particularly in immunocompromised hosts. However, their clinical epidemiology and antifungal management data remain limited. **Methods**: We conducted a retrospective study of patients with filamentous basidiomycetes isolated from clinical specimens at King Chulalongkorn Memorial Hospital, Thailand, between 2019 and 2025. Species identification was performed using internal transcribed spacer (ITS) or D1/D2 ribosomal DNA sequencing. Demographic characteristics, clinical features, antifungal management, and outcomes were analyzed. **Results**: Fourteen patients were identified with a mean age of 61.2 ± 18.3 (29–90), and 71.4% were female. In these patients, pulmonary infection was most common (64.3%), followed by ocular (14.3%), cutaneous (14.3%), and central-line-associated infection (7.1%). Bronchoalveolar lavage was the most frequent specimen (64.3%). ITS/D1D2 sequencing revealed broad species diversity, including *Schizophyllum commune* (n = 3), *Candolleomyces* spp. (n = 3), *Coprinopsis cinerea*, *Fomitopsis* spp., *Geliporus exilisporus*, *Odontoefibula orientalis*, *Irpex laceratus*, *Volvariella volvacea*, *Deconica coprophila*, and *Agaricales* spp. Antifungal therapy was largely empirical, with voriconazole used most frequently (46.6%). Overall, 85.7% of patients improved, whereas 14.3% did not respond clinically. **Conclusions**: Emerging filamentous basidiomycetes demonstrate substantial species diversity and pose ongoing diagnostic and antifungal management challenges. The absence of standardized susceptibility testing and clinical breakpoints may contribute to therapeutic uncertainty and challenges in antifungal selection. Integrating molecular diagnostics into routine clinical workflows may enhance antifungal stewardship in rare mold infections.

## 1. Introduction

Invasive and chronic respiratory fungal diseases remain under-recognized worldwide and are associated with substantial morbidity and mortality, particularly among immunocompromised individuals [1]. Although antifungal resistance is most intensively studied in *Candida* and *Aspergillus*, the expanding spectrum of filamentous fungi implicated in human diseases underscores the need to reassess resistance beyond traditional pathogens. Environmental azole exposure and clinical antifungal pressure have contributed to increasing filamentous fungal resistance, complicating therapeutic strategies and outcomes [1]. Filamentous basidiomycetes are increasingly recognized as opportunistic human pathogens capable of causing allergic, chronic, and invasive infections, most frequently involving the respiratory tract [2,3,4]. Among these organisms, *Schizophyllum commune* is the most frequently reported species in human disease and has been implicated in allergic bronchopulmonary mycosis, chronic pulmonary infection, fungus ball formation, and invasive disease [4,5]. *Coprinopsis cinerea*, formerly known as *Hormographiella aspergillata*, is another clinically important basidiomycete that has been associated with severe pulmonary and disseminated infection, particularly in profoundly immunocompromised patients [4,6,7]. By contrast, other basidiomycetes such as *Volvariella* spp. and related agaricoid fungi remain rarely reported in human specimens, and their pathogenic roles are often uncertain because of the limited number of published cases and incomplete clinicopathologic correlation [4,6].

A major barrier to accurate recognition is diagnostic difficulty, as clinical basidiomycete isolates frequently appear as white, cottony, nonsporulating molds that lack distinctive microscopic structures under routine laboratory conditions [2,4]. Such isolates are often reported as “sterile molds” or remain unidentified, which may delay appropriate antifungal therapy and complicate clinical relevance assessment [2,4]. In this context, molecular identification methods, particularly sequencing of the internal transcribed spacer (ITS) and large subunit (LSU) rDNA regions (D1/D2), have become essential tools for species-level identification of nonsporulating molds [2,5].

In a global review of 218 reported cases, it was demonstrated that *Schizophyllum commune* accounted for approximately half of human infections, followed by *Coprinopsis cinerea*, *Emmia lacerata*, and other rare taxa, highlighting the growing clinical diversity of this group [4]. Despite this expanding recognition, regional epidemiologic data remain limited, particularly in Asia, and the true disease burden is likely underestimated [2,3].

Therapeutic management of filamentous basidiomycetes is further complicated by the absence of standardized antifungal susceptibility testing methods and established clinical breakpoints, limiting minimum inhibitory concentration interpretation in routine practice [2,5].

Available in vitro data suggest that many basidiomycetes exhibit high MICs to fluconazole and limited echinocandin activity, whereas amphotericin B and mold-active triazoles such as itraconazole, voriconazole, posaconazole, and isavuconazole generally demonstrate lower MICs. However, susceptibility patterns remain species-dependent and incompletely characterized [1,2,5,8]. For example, clinical isolates of *S. commune* show low geometric mean MICs for amphotericin B and triazoles but high MICs for fluconazole and flucytosine, illustrating heterogeneity in antifungal susceptibility within this group [5]. Beyond intrinsic susceptibility profiles, delayed species recognition and empirical antifungal therapy may contribute to challenges in antifungal selection, in which diagnostic uncertainty, inappropriate initial therapy, or lack of susceptibility benchmarks adversely influence clinical outcomes [2,8]. Such challenges are particularly relevant in tertiary-care centers managing transplant recipients, hematologic malignancies, and other high-risk populations, where prompt and accurate antifungal therapy is critical.

Given these diagnostic and therapeutic uncertainties, systematic clinical data from under-reported tropical regions are needed to clarify the epidemiology, antifungal management strategies, and outcomes of infections caused by filamentous basidiomycetes [2,4].

In this study, we describe the clinical characteristics, molecular identification, antifungal treatment approaches, and outcomes of patients with filamentous basidiomycetes isolated from clinical specimens at a tertiary-care center in Thailand, emphasizing diagnostic and antifungal challenges relevant to the broader landscape of antifungal resistance.

## 2. Results

### 2.1. Fourteen Patients with Emerging Filamentous Basidiomycetes Were Identified in a High-Risk Tertiary-Care Population

A total of 14 patients with filamentous basidiomycetes isolated from clinical specimens were identified between 2019 and 2025 (Table 1). The mean age was 61.2 ± 18.3 years (range 29–90), and 71.4% were female. Underlying conditions included end-stage renal disease with kidney transplantation, hematologic malignancy (AML, MDS) with hematopoietic stem cell transplantation, systemic lupus erythematosus, systemic sclerosis, interstitial lung disease, bronchiectasis, hepatocellular carcinoma, and solid malignancy, reflecting a population with substantially immunocompromised or structural lung disease-infected individuals.

### 2.2. Pulmonary Infection Was the Predominant Clinical Presentation

Pulmonary involvement accounted for 64.3% (9/14) of cases, followed by ocular (14.3%, 2/14), cutaneous (14.3%, 2/14), and central-line-associated infections (7.1%, 1/14) (Table 1). Bronchoalveolar lavage was the most common specimen (64.3%), followed by ocular (14.3%), tissue/pus (14.3%), and other specimens (7.1%) (Table 1). These data underscore the respiratory tract as the primary clinical site of basidiomycete isolation in this cohort.

### 2.3. Conventional Microscopy and Cultures Lacked Species-Level Resolution

**Figure 1:**
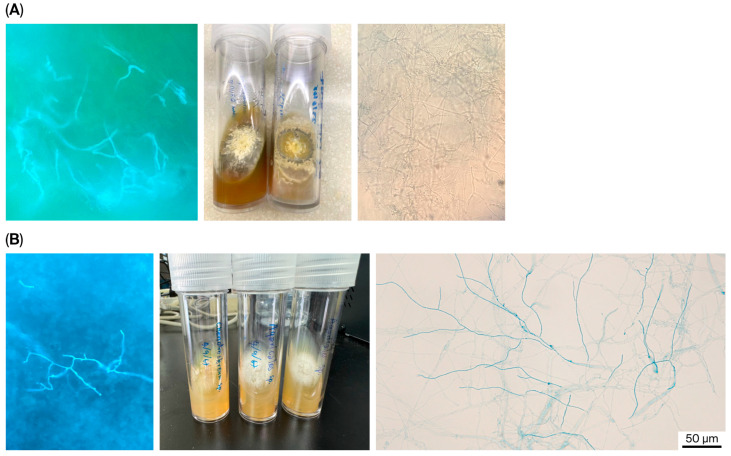
Representative morphologic features of filamentous Basidiomycota isolated from clinical specimens. (**A**) Corneal scraping isolate from patient no. 1 identified as *Candolleomyces rubrobrunneus*. Left: direct microscopy with calcofluor white (CFW) demonstrating hyaline septate hyphae; middle: colony morphology in culture; right: microscopic morphology (lactophenol cotton blue; LCB wet mount) from culture preparation showing hyaline hyphae with non-distinctive features on light microscopy. (**B**) Skin biopsy specimen from patient no. 3 identified as *Agaricales* spp. Left: direct microscopy with CFW demonstrating hyaline septate hyphae; middle: colony morphology in culture; right: microscopic morphology (LCB wet mount) from culture preparation showing hyaline, septate hyphae. (Scale bars as indicated, if applicable).

Direct microscopic findings varied according to clinical syndrome and specimen type: hyaline septate hyphae were observed in ocular and cutaneous specimens, whereas darkly pigmented septate hyphae were noted in a trauma-associated ocular infection. In several pulmonary episodes, direct microscopy was negative despite subsequent culture positivity. Culture reports frequently lacked species-level resolution and were described as “nonsporulating septate hyphae” or “unidentified mold,” highlighting the limited discriminatory capacity of conventional morphology for filamentous basidiomycetes (Figure 1).

Histopathological examination was not systematically performed for all cases but was available only when tissue specimens were obtained through routine clinical care, typically in patients with suspected invasive infection requiring biopsy or surgical sampling. In these cases, histopathologic findings, when present, demonstrated tissue invasion and associated host inflammatory responses. A kidney transplant recipient with cutaneous infection showed granulomatous inflammation, while a pulmonary case exhibited non-necrotizing granuloma. Notably, one HSCT recipient (patient no. 11) demonstrated broad, pauciseptate ribbon-like hyphae in tissue sections, morphologically resembling mucormycetes, illustrating significant morphologic overlap among invasive molds in immunocompromised hosts and underscoring the diagnostic limitations of morphology-based identification, which may contribute to inappropriate empirical antifungal selection and delayed species-directed therapy. In this case, the initial morphologic impression suggested mucormycosis, prompting empirical treatment with amphotericin B; however, after molecular identification confirmed a filamentous basidiomycete, antifungal therapy was adjusted to voriconazole, reflecting the revised diagnostic interpretation.

### 2.4. ITS and D1/D2 Sequencing Revealed Broad Taxonomic Diversity

Molecular identification demonstrated considerable species heterogeneity (Table 2). *Schizophyllum commune* was identified in three cases, and *Candolleomyces* spp. (including *C. rubrobrunneus*) were identified in another three. Other identified organisms included *Coprinopsis cinerea*, *Fomitopsis* spp., *Geliporus exilisporus*, *Odontoefibula orientalis*, *Irpex laceratus*, *Volvariella volvacea*, *Deconica coprophila*, and *Agaricales* spp, with sequence identity ranging from 97.54% to 100% with high query coverage, supporting robust molecular identification (Supplementary Table S1).

### 2.5. Antifungal Therapy Was Largely Empirical and Clinical Non-Improvement Occurred in a Measurable Proportion of Cases

Voriconazole was the most frequently prescribed antifungal agent, administered in seven of fourteen patients (46.6%). Posaconazole was used in one patient (6.7%), while another (6.7%) received combination therapy with liposomal amphotericin B plus voriconazole. Six patients (40%; patient no. 2, 7, 9, 10, 13, and 14) did not receive antifungal therapy because the isolates were considered possible colonizers or contaminants and treatment decisions were guided by overall clinical assessment rather than microbiologic findings alone. Voriconazole was administered at a total daily dose of 200–500 mg for a minimum duration of 12 weeks in treated cases.

Overall, 12 of 14 patients (85.7%) demonstrated clinical improvement based on physician-documented clinical course, supported by available radiologic and/or histopathologic data, whereas 2 patients (14.3%; patients no. 2 and 5) did not (Supplementary Figure S1). These included one case of untreated pulmonary *Coprinopsis cinerea*, in which clinical deterioration was attributed to complications of the underlying disease, and one case of pulmonary *Schizophyllum commune* infection treated with voriconazole, which showed radiographic progression on chest X-ray (Supplementary Figure S1) (Table 3). The occurrence of clinical non-response despite mold-active therapy, together with the absence of antifungal susceptibility testing, highlights the therapeutic uncertainty associated with filamentous basidiomycetes and the potential contribution of species-specific intrinsic resistance or suboptimal antifungal selection.

## 3. Discussion

Filamentous basidiomycetes are increasingly recognized as opportunistic human pathogens, particularly in immunocompromised hosts, although they remain under-reported in routine clinical practice [2,4]. Most reported cases involve respiratory disease, reflecting the ecological predominance of these organisms in environmental reservoirs and their inhalational exposure route [1,2,3]. Our finding that pulmonary infection was the most common presentation is therefore consistent with previous clinical summaries and global case reviews [1,2,3,4].

Despite increasing recognition, conventional diagnostic approaches remain limited, as many basidiomycete isolates present as sterile, white, nonsporulating molds that lack distinctive microscopic features under standard laboratory conditions [1,9]. This frequently results in inconclusive culture reports and delayed species-level identification, which may affect antifungal selection and clinical outcomes [1,5,9]. Morphological overlap with other invasive molds, particularly in immunocompromised hosts, further complicates syndromic diagnosis and may lead to inappropriate empirical therapy [2,4,5,8].

Molecular identification using ITS sequencing has become the most practical and widely accepted method for identifying nonsporulating molds and rare filamentous fungi in clinical laboratories [2,5], and revealed substantial species diversity beyond commonly recognized pathogens such as *Schizophyllum commune* in our cohort, reflecting the expanding spectrum of clinically relevant basidiomycetes. However, limitations in reference sequence databases and incomplete taxonomic representations may reduce discriminatory accuracy in certain genera, underscoring the importance of high-quality sequence analysis and, when feasible, multi-locus confirmation [2,5].

From an antifungal resistance perspective, therapeutic uncertainty arises not only from intrinsic or acquired resistance mechanisms but also from diagnostic delay and the absence of interpretive susceptibility breakpoints for rare molds [5,6,10]. Unlike *Candida* and *Aspergillus*, most filamentous basidiomycetes lack CLSI or EUCAST clinical breakpoints, limiting the translation of MIC data into evidence-based treatment decisions [6,10].

Importantly, in clinical practice, treating filamentous basidiomycete infections generally centers on mold-active triazoles, particularly itraconazole and voriconazole, with amphotericin B as an alternative in selected cases [2,5,11]. In contrast, fluconazole and flucytosine are not considered standard therapeutic agents for these organisms due to their limited activity [2,5]. Consistent with this, available in vitro data suggest that many basidiomycetes exhibit high MICs to fluconazole and flucytosine and limited susceptibility to echinocandins, whereas amphotericin B and mold-active triazoles (itraconazole, voriconazole, posaconazole, isavuconazole) typically demonstrate comparatively lower MICs and are therefore more clinically relevant options [2,5,11].

For example, susceptibility profiling of *S. commune* isolates demonstrated low geometric mean MICs for amphotericin B and triazoles but elevated MICs for fluconazole and flucytosine, highlighting species-dependent variability in antifungal response [9]. Similarly, *Hormographiella aspergillata* (the anamorph of *Coprinopsis cinerea*) has been associated with reduced echinocandin susceptibility and breakthrough infections during caspofungin therapy, raising concerns regarding empirical echinocandin monotherapy in certain basidiomycete infections [7].

This pattern is also supported by data from *Ceriporia lacerata* respiratory isolates, in which low MICs were reported for posaconazole and isavuconazole (0.06–0.125 μg/mL), itraconazole (0.06–0.5 μg/mL), voriconazole (0.125–0.5 μg/mL), and amphotericin B (0.25–1 μg/mL), whereas echinocandins showed high MEC values (8 μg/mL), further suggesting that mold-active triazoles and amphotericin B may be more active than echinocandins against certain filamentous basidiomycetes [9].

However, interpreting these susceptibility data remains challenging because there are currently no CLSI or EUCAST clinical breakpoints and no robust epidemiological cutoff values for most basidiomycetes. As a result, published studies generally report raw MIC or MEC values generated using different antifungal panels, testing conditions, and small isolate numbers, which limits direct comparison of susceptibility versus resistance across reports [5,9,11,12].

Although antifungal susceptibility testing was not available in our cohort, published data on *Schizophyllum commune* provide important contextual insight. In a study of 30 clinical isolates using CLSI methodology, low geometric mean MICs were reported for amphotericin B (0.29 µg/mL), itraconazole (0.20 µg/mL), and voriconazole (0.24 µg/mL) [5]. More recently, a large multicenter study evaluating 113 isolates using both CLSI and EUCAST methods demonstrated consistently low MICs for amphotericin B and voriconazole across both platforms, although notable inter-method variability was observed for other azoles, with EUCAST MICs generally higher than CLSI values by approximately two dilution steps [11]. These findings highlight the methodological variability and lack of standardized breakpoints for filamentous basidiomycetes, which complicate the interpretation of MIC data across studies. Despite these inconsistencies, both CLSI- and EUCAST-based data consistently identify mold-active triazoles and amphotericin B as the most active agents in vitro. This is in line with our clinical observations, where voriconazole was the most frequently used antifungal agent and was associated with overall clinical improvement in the majority of treated cases. Taken together, these data suggest that, even in the absence of isolate-specific MIC testing, current evidence supports the use of mold-active triazoles, particularly voriconazole, as a reasonable first-line therapeutic approach for *S. commune* infections. However, the observed variability between susceptibility testing methodologies underscores the need for standardized testing protocols and prospective studies integrating MIC data with clinical outcomes.

Accordingly, the most meaningful comparison to make across studies is often the relative susceptibility pattern rather than formal categorical interpretation. Overall, the available literature supports a general preference for mold-active triazoles, including itraconazole, voriconazole, posaconazole, and isavuconazole, or amphotericin B for invasive disease, while fluconazole and echinocandins appear less reliable as monotherapy except in selected circumstances where alternative agents are not available or when the clinical context suggests colonization, allergic disease, or another noninvasive presentation [5,7,9,11,12].

In our cohort, antifungal therapy was largely empirical, with voriconazole most frequently prescribed, reflecting current clinical practice for invasive mold infections [6,10]. For a case with morphologic features mimicking mucormycetes on direct microscopy, the initial impression led to empirical treatment with amphotericin B, consistent with standard management of suspected mucormycosis. Following molecular identification, antifungal therapy was subsequently modified to voriconazole, reflecting the revised etiologic diagnosis. Although amphotericin B may retain activity against some basidiomycetes, this example highlights how diagnostic uncertainty may influence early antifungal selection and potentially delay the use of mold-active triazoles, thereby contributing to therapeutic uncertainty in clinical practice.

Although a broad diversity of filamentous basidiomycete taxa was identified in this cohort, not all isolates were considered clinically pathogenic. Based on a clinicomicrobiologic assessment informed by EORTC/MSGERC criteria [13], five of fourteen cases (35.7%) were classified as proven IFD (cases 1, 3, 4, 11, and 12), three of fourteen (21.4%) as probable IFD (cases 5, 6, and 8), and six of fourteen (42.9%) as having uncertain clinical significance, including possible colonization or contamination (cases 2, 7, 9, 10, 13, and 14) (Table 3).

Pathogenic cases in this cohort were associated with a range of filamentous basidiomycetes, including *Candolleomyces* spp. (n = 3), *Schizophyllum commune* (n = 2), *Agaricales* spp. (n = 1), *Geliporus exilisporus* (n = 1), and *Volvariella volvacea* (n = 1), when identified in appropriate clinical contexts supported by host factors and clinicoradiologic or histopathologic findings [13]. In contrast, isolates of *Coprinopsis cinerea*, *Fomitopsis* spp., *Odontoefibula orientalis*, *Irpex laceratus*, and *Deconica coprophila* were more frequently classified as having uncertain clinical significance, including possible colonization or contamination, particularly when recovered from respiratory specimens without supportive clinical or radiologic evidence.

These findings underscore the importance of cautious basidiomycete isolation interpretation, especially from nonsterile sites, where organism recovery does not necessarily equate to invasive infection. Integrating host factors, clinical presentation, radiologic findings, and supportive microbiologic or histopathologic evidence is therefore essential to distinguish true infection from colonization and to guide appropriate antifungal management.

Although most evaluable patients improved, clinical non-response occurring in a subset of cases illustrates how therapeutic uncertainty may contribute to what can be conceptualized as challenges in antifungal selection, even in the absence of documented in vitro resistance [2,5]. In particular, the occurrence of clinical non-improvement in selected cases in this cohort may reflect factors beyond intrinsic resistance, including delayed diagnosis, variability in antifungal exposure, or unrecognized differences in species-specific susceptibility. Notably, 40% of isolates were ultimately considered colonization or contamination and these patients experienced clinical improvement without antifungal therapy. This observation underscores the importance of careful clinical correlation when interpreting mold isolation, particularly for rare basidiomycetes. Over-treatment may expose patients to unnecessary drug toxicity, whereas under-recognition of invasive disease may delay appropriate therapy; therefore, optimal management requires integrating clinical judgment, radiologic and histopathologic findings, molecular identification, and, when available, antifungal susceptibility testing. This framework highlights that resistance in clinical mycology extends beyond target-gene mutations and acquired MIC elevation to include diagnostic ambiguity, delayed species-level identification, and the absence of standardized susceptibility interpretation for rare molds [2,5].

From a clinical management perspective, the decision to initiate antifungal therapy depends on both the isolation site and the clinical context. Treatment is most clearly indicated when filamentous basidiomycetes are recovered from normally sterile sites (e.g., blood, tissue biopsy, or central-line-associated specimens) or when there is histopathologic evidence of tissue invasion, as these findings are consistent with proven invasive fungal disease [13].

In pulmonary infections, antifungal therapy should be considered when isolation occurs in patients with relevant host risk factors (e.g., hematologic malignancy or transplantation) accompanied by compatible clinical symptoms and progressive radiologic abnormalities, particularly when alternative etiologies have been excluded [6,13]. For extrapulmonary sites such as ocular or cutaneous infections, treatment is generally indicated when there is localized tissue involvement with supportive clinical or histopathologic evidence, especially in immunocompromised hosts [2,4]. In contrast, in the absence of supportive clinicoradiologic or histopathologic findings, isolation from nonsterile sites, particularly respiratory specimens such as bronchoalveolar lavage, is more likely to represent colonization or contamination and may not warrant antifungal therapy [2,4,8]. In such cases, close clinical monitoring is recommended.

Our study has several limitations: First, its retrospective single-center design limited the completeness and uniformity of the clinical data collection, including the histopathologic and morphologic images. In particular, radiologic findings, detailed histopathologic descriptions, and standardized longitudinal follow-up assessments were not consistently available for all patients, especially given that most isolates were recovered from pulmonary specimens. As this study was conducted in a tertiary-care referral center, a proportion of patients were initially evaluated and followed at primary or referring hospitals. Consequently, some radiologic data—including baseline imaging and serial follow-up studies—were not available in digital format or could not be retrieved due to loss of original records. This limitation restricted the ability to perform standardized longitudinal or semi-quantitative radiologic assessments across all cases and contributed to reliance on descriptive interpretation. Consequently, clinical improvement could not be defined using a single uniform endpoint such as radiologic resolution, culture clearance, or negative fungal PCR; instead, outcomes were assessed retrospectively based on the overall clinical judgment of the physician treating the infectious diseases, supported by available clinical, radiologic, microbiologic, and histopathologic data when present. Second, the small sample size limited statistical power and generalizability to other settings. Third, antifungal susceptibility testing was not systematically performed, and minimum inhibitory concentration (MIC) data were therefore unavailable. This was partly due to resource limitations at the time of sample collection, which precluded the establishment of a structured mycology strain repository; consequently, viable isolates were not preserved for retrospective susceptibility testing. In addition, even when MIC data are available, their clinical interpretation for filamentous basidiomycetes remains challenging. There is significant inter-method variability between CLSI and EUCAST methodologies, with reported differences of up to two dilution steps for certain antifungal agents, and no standardized clinical breakpoints or robust epidemiological cutoff values currently exist for most basidiomycetes [5,11]. The absence of isolate-specific MIC data in our study limited the ability to assess species-specific susceptibility variability and to correlate in vitro antifungal activity with clinical outcomes. This may contribute to therapeutic uncertainty, particularly in cases of clinical non-improvement despite mold-active therapy, and reinforces the need for integrated clinicomicrobiologic interpretation rather than reliance on susceptibility data alone.

From a clinical perspective, these limitations highlight that antifungal decision-making in filamentous basidiomycete infections currently relies heavily on empirical therapy and clinical judgment. While mold-active triazoles such as voriconazole are generally preferred based on available in vitro data, the lack of standardized susceptibility frameworks may lead to suboptimal antifungal selection in certain cases. This underscores the importance of combining clinical, radiologic, microbiologic, and molecular data to guide treatment decisions.

Our future direction would be the establishment of a multicenter cohort or collaborative registry involving tertiary-care hospitals caring for high-risk patient populations to enhance case capture and improve external validity. Such an approach would enable standardized case definitions, harmonized clinical outcome measures, and more robust comparative analyses across species, infection syndromes, and antifungal treatment strategies. In addition, the systematic incorporation of antifungal susceptibility testing, integrated with pharmacokinetic–pharmacodynamic analyses, would facilitate more meaningful correlations between microbiologic findings and clinical outcomes, thereby strengthening the evidence base for antifungal selection in these rare pathogens.

Radiologic assessment in the present study was not standardized across cases. Serial imaging and comparable radiologic datasets were not consistently available for all patients, particularly among pulmonary cases, limiting the ability to perform uniform or semi-quantitative evaluation of disease progression (e.g., lesion size variation, density changes, or standardized scoring approaches). Consequently, radiologic outcomes were interpreted descriptively rather than using predefined objective criteria. For similar reasons, comprehensive imaging for all 14 patients could not be included, as complete and comparable imaging datasets were not uniformly available. Inclusion of heterogeneous or incomplete imaging series could introduce interpretative bias and limit meaningful comparison across cases. Therefore, representative imaging was selected to illustrate typical patterns of radiologic response.

Future studies should incorporate standardized imaging protocols with predefined time points, uniform acquisition parameters, and centralized image review. The application of semi-quantitative or quantitative radiologic metrics, including lesion measurement, volumetric assessment, or density-based scoring systems, would enable more objective evaluation of treatment response and facilitate correlation with microbiologic findings and clinical outcomes. Prospective multicenter studies will be essential to validate these approaches and to better define the clinical relevance of minimum inhibitory concentration (MIC) variability within species.

Despite these limitations, our study integrates molecular identification with detailed clinical characterization and provides region-specific insights into the diagnostic and therapeutic challenges posed by emerging filamentous basidiomycetes in a tropical tertiary-care setting.

## 4. Materials and Methods

### 4.1. Study Design and Setting

We conducted a retrospective observational study at King Chulalongkorn Memorial Hospital (KCMH), a tertiary-care center in Bangkok, Thailand. Clinical specimens collected between 2019 and 2025, yielding molds identified as filamentous basidiomycetes by molecular methods, were eligible for inclusion. Only isolates with available molecular identification were included; specifically, inclusion required internal transcribed spacer (ITS) sequencing with Sanger-based confirmation of Basidiomycota. Isolates without sequencing data were excluded from the analysis. We reported this in accordance with the STROBE guidelines for observational cohort studies (Supplementary File S1).

### 4.2. Case Identification and Inclusion Criteria

The clinical significance of each isolate was assessed using clinicomicrobiologic criteria informed by the 2020 revised definitions of invasive fungal disease (IFD) from the European Organization for Research and Treatment of Cancer/Mycoses Study Group Education and Research Consortium (EORTC/MSGERC) [13].

Cases were classified as proven, probable, or possible IFD. Proven IFD was defined by histopathologic or cytopathologic evidence of fungal elements in damaged tissue, or recovery of the organism from a normally sterile site. Probable IFD required the presence of at least one host factor, compatible clinical features (including radiologic findings), and mycological evidence. Possible IFD was defined by the presence of host factors and compatible clinical features without mycological confirmation.

Because filamentous basidiomycetes are frequently isolated from nonsterile sites, particularly respiratory specimens, and may represent colonization, environmental contamination, or transient airway presence, all cases were further evaluated using clinicomicrobiologic correlation. This included assessing host risk factors, compatible clinical and radiologic findings, repeated isolation results when available, and supportive microbiologic or histopathologic evidence [13]. Cases that did not meet the criteria for proven, probable, or possible IFD, or lacked sufficient supporting evidence, were classified as having uncertain clinical significance, including possible colonization or contamination.

A summary of the case definitions and classification criteria used in this study is provided in Table 3. Given that EORTC/MSGERC definitions were developed primarily for immunocompromised populations and for research standardization, their applicability to all patient groups and to rare molds such as filamentous basidiomycetes may be limited, and findings were interpreted accordingly [13].

Clinical outcomes were assessed retrospectively based on physician-documented clinical course, including improvements in symptoms, radiologic findings when available, and overall disease trajectory; no single standardized endpoint was applied.

### 4.3. Conventional Mycology Workflow (Direct Examination and Culture Reporting)

Direct microscopic examination results were extracted from the clinical record/case table. Culture results were recorded as originally reported by the clinical laboratory (e.g., “nonsporulating septate hyphae,” “unidentified mold,” or a genus/species name when available).

### 4.4. Molecular Identification (ITS and D1/D2 Sequencing and Taxonomic Assignment)

Species identification was performed using PCR targeting of the internal transcribed spacer (ITS) and D1/D2 regions with Sanger sequencing, as previously described [14,15]. Purified PCR products were sequenced using the BigDye Terminator v3.1 Cycle Sequencing Kit and analyzed on an ABI 3730xl Genetic Analyzer (Applied Biosystems, Thermo Fisher Scientific, Waltham, MA, USA).

Sequence data were compared against the National Center for Biotechnology Information (NCBI) GenBank database using BLASTn (https://blast.ncbi.nlm.nih.gov) for taxonomic assignment. Species-level identification was assigned when the sequence identity was ≥99%, while identities between 97% and 98.9% were considered sufficient for genus-level identification. Sequences with <97% identity were reported at higher taxonomic levels or considered inconclusive.

Sequence-based identification outputs, including organism name, percent identity, and query coverage, are summarized in Table 2 and Supplementary Table S1. Corresponding GenBank accession numbers are provided in Supplementary Table S1.

### 4.5. Statistical Analysis

Variables were summarized descriptively as mean ± SD (range) for continuous data and n (%) for categorical data.

### 4.6. Ethics Statement

This study was approved by the Institutional Review Board (IRB No. 601/67, COE No. 047/2024, approved on 30 July 2024) of the Faculty of Medicine, Chulalongkorn University, Bangkok, Thailand.

## 5. Conclusions

Filamentous basidiomycetes represent an under-recognized but clinically relevant group of opportunistic molds capable of causing pulmonary and extrapulmonary infections in high-risk populations. Conventional morphology-based diagnostics frequently lack sufficient discriminatory power, whereas ITS sequencing enables definitive identification and reveals broader species diversity than previously appreciated. In the absence of standardized susceptibility testing and clinical breakpoints, antifungal therapy remains largely empirical, and measurable clinical non-improvement rates underscore the stewardship implications of diagnostic delay.

Future research should prioritize multicenter surveillance, standardized antifungal susceptibility methodologies, improved sequence database curation, and the integration of molecular diagnostics into antifungal resistance frameworks to better guide the treatment of these emerging pathogens.

## Figures and Tables

**Table 1:** Demographic data of 14 patients with Basidiomycota fungi infection at King Chulalongkorn Memorial Hospital from 2019 to 2025.

Characteristics	Number of Patients, n (%)
Age (years), mean ± SD (min–max)	61.2 ± 18.3 (29–90)
Sex	
Male	4 (28.6%)
Female	10 (71.4%)
Area of infection	
Pulmonary	9 (64.3%)
Ocular	2 (14.3%)
Skin	2 (14.3%)
Central line	1 (7.1%)
Specimens	
Bronchoalveolar lavage	9 (64.3%)
Ocular specimens	2 (14.3%)
Tissue/Pus	2 (14.3%)
Others	1 (7.1%)
Treatment	
Voriconazole	7 (46.6%) *
None	6 (40%)
Posaconazole	1 (6.7%)
Liposomal amphotericin B	1 (6.7%) *
Outcome	
Improved	12 (85.7%)
Not improved	2 (14.3%)

* One patient received combination therapy with liposomal amphotericin B and voriconazole. Percentage calculated from N = 14.

**Table 2:** Molecular identification of filamentous basidiomycetes by ITS or D1/D2 sequencing, including sequence identity and query coverage from NCBI database (* colonization/contamination).

No.	Sequencing Information			
	Identification	Identity (%)	Query Coverage (%)	ITS or D1/D2
1	*Candolleomyces* spp.	99.5	100	ITS
2	*Coprinopsis cinerea* *	100	100	ITS
3	*Agaricales* spp.	99.64	100	D1/D2
4	*Candolleomyces* spp.	97.58	100	ITS
5	*Schizophyllum commune*	100	100	ITS
6	*Schizophyllum commune*	100	100	ITS
7	*Fomitopsis* spp. *	100	100	ITS
8	*Geliporus exilisporus*	99.09	100	ITS
9	*Odontoefibula orientalis* *	98.24	100	ITS
10	*Irpex lacerates* *	99.82	100	ITS
11	*Volvariella volvacea*	100	100	D1/D2
12	*Candolleomyces rubrobrunneus*	100	99.66	ITS
13	*Schizophyllum commune* *	100	100	D1/D2
14	*Deconica coprophila* *	100	99.82	ITS

**Table 3:** Case definitions and classification of filamentous basidiomycete isolates based on EORTC/MSGERC criteria and clinicomicrobiologic correlation of each patient with Basidiomycota fungi infections at King Chulalongkorn Memorial Hospital during 2019–2025.

Case No.	Year	Age (Years)	Sex	Underlying Disease	Site of Infection	Fungal Culture Result	EORTC/MSG Definition	Treatment	Outcome
1	2019	90	Male	Hepatocellular carcinoma	Ocular	Nonsporulating septate hyphae	Proven	Voriconazole	Improved
2	2021	70	Female	SLE	Pulmonary	Nonsporulating septate hyphae	Colonization/contamination	None	Progression from imaging
3	2023	66	Female	ESRD with kidney transplant	Skin	Nonsporulating septate hyphae	Proven	Posaconazole	Improved
4	2023	38	Female	ESRD with kidney transplant	Central line	Nonsporulating septate hyphae	Proven	Voriconazole	Improved
5	2023	47	Female	ESRD	Pulmonary	Nonsporulating septate hyphae with clamp connection	Probable	Voriconazole	Progression from imaging
6	2023	46	Female	AML s/p allogeneic HSCT	Pulmonary	Nonsporulating septate hyphae with clamp connection	Probable	Voriconazole	Improved
7	2024	77	Female	Interstitial lung disease	Pulmonary	Nonsporulating septate hyphae	Colonization/contamination	None	Improved
8	2024	76	Male	Bronchiectasis	Pulmonary	Nonsporulating septate hyphae	Probable	Voriconazole	Improved
9	2024	77	Female	Bronchiectasis	Pulmonary	Nonsporulating septate hyphae	Colonization/contamination	None	Improved
10	2024	29	Female	SLE	Pulmonary	Nonsporulating septate hyphae	Colonization/contamination	None	Improved
11	2025	62	Male	MDS with allogeneic HSCT	Skin	Negative	Proven	Liposomal amphotericin B, Voriconazole	Improved
12	2025	41	Male	Lacrimal sac tumor	Ocular	Nonsporulating septate hyphae	Proven	Voriconazole	Improved
13	2025	78	Female	Lung cancer with lobectomy	Pulmonary	Nonsporulating septate hyphae with clamp connection	Colonization/contamination	None	Improved
14	2025	60	Female	systemic sclerosis	Pulmonary	Nonsporulating septate hyphae	Colonization/contamination	None	Improved

SLE = systemic lupus erythematosus; ESRD = end-stage renal disease; AML = acute myeloblastic leukemia; HSCT = hematopoietic stem cell transplantation; MDS = myelodysplastic syndrome.

## Data Availability

All data used to support the findings of this study are included within the article and the raw data for each figure/table are available from the corresponding author upon request.

## Supplementary Materials

The following supporting information can be downloaded at: https://www.mdpi.com/article/10.3390/antibiotics15050424/s1, Supplementary File S1: STROBE Statement—Checklist of items that should be included in reports of cohort studies. Supplementary Table S1: Molecular identification of filamentous basidiomycetes by ITS/D1D2 sequencing, including organism name, sequence, and base pairs. Supplementary Figure S1: Contrast-enhanced chest CT images of patient no. 6 before treatment (A) and after treatment (B) demonstrate radiologic improvement. Contrast-enhanced chest CT images of patient no. 13 before treatment (C) and after treatment (D) also demonstrate radiologic improvement. In contrast, chest radiographs of patient no. 5 obtained prior to treatment (E) and following treatment (F) demonstrate radiologic progression.
