# Supplementary material for: Emerging Filamentous Basidiomycetes as Opportunistic Human Pathogens: Diagnostic and Antifungal Challenges in a Tertiary-Care Center in Thailand
Source: Antibiotics (Basel). 2026 Apr 23;15(5):424. doi: 10.3390/antibiotics15050424 (PMC13203384; doi:10.3390/antibiotics15050424)
Supplement: Supplementary file 1 [file antibiotics-15-00424-s001.zip › antibiotics-4195909-supplementary.pdf]

Supplementary Table S1. Molecular identification of filamentous basidiomycetes by ITS/D1D2 sequencing, including organism name, sequence, base pairs, and NCBI accession number, is demonstrated.

No	Organism	sequence	ITS/D1D2	Base pairs	Accession number
1	*Candolleomyces* spp.	GGCTCTTAGGAGCATTTGTGCACGCCCGTCATTCATATCACCTTTCCACCTGTGAA<br>CTCTGTGTAGGCCTGGATAACCCTCGCTTTGGCAACAAAGCGGATGCAGGGATTG<br>CTGTGTCGACAAGGCCAGCTCTCCTTGAATTTCCAGGTTCTATGTCTTTTTACACA<br>CCCCATTTGAATGATGTAGAATGTAGTCAATGGGCTTTCATGCCTATAAAACACTA<br>TACAACTTTCAGCAACGGATCTCTTGGCTCTCGCATCGATGAAGAACGCAGCGAA<br>ATGCGATAAGTAATGTGAATTGCAGAATTCAGTGAATCATCGAATCTTTGAACGC<br>ACCTTGCGCTCCTTGGTATTCCGAGGAGCATGCCTGTTTGAGTGTCATTAAATTCT<br>CAACCTCACCAGTTTTGTAACGAAACGGGTGAAGGCTTGGATGTGGGGGCTTTGC<br>AGGCTGCCTTAGTGCTGGTCTGCTCCCCTGAAATGCATTAGCGAGCTCATACTGAG<br>CTTCCGTCTATTGGTGTGATAATTATCTACGCCGTGGATTGGACTCTGTGCTTGCTT<br>CTAACCGTCCGCAAGGACAATCTACTTGACCAATTTGACCTCA	ITS	600	PX843587.1
2	*Coprinopsis cinerea*	AGCATGTGCACGCCGGTCACCTTTATTTCTCCACCTGTGCACACACTGTAGGCCTG<br>GATACCTCTCGTCGCAAGGCGGATGCGTGGCTTGCTGTCGCTTTCGAAAGAAGGC<br>CGGCTTGCCATGAATTTCCAGGTCTATGATTTCTTACACACCCCAAACTGAATGTT<br>ATGGAATGTCATCTCAAGGCCTTGTGCCTATAAACCTATACAACTTTCAGCAACGG<br>ATCTCTTGGCTCTCGCATCGATGAAGAACGCAGCGAAATGCGATAAGTAATGTGA<br>ATTGCAGAATTCAGTGAATCATCGAATCTTTGAACGCACCTTGCGCTCCTTGGTAT<br>TCCGAGGAGCATGCCTGTTTGAGTGTCATTAAATTCTCAACCTCACCAACTTTGTT<br>GTGTGCAGGCTTGGATGTGGGGGTTGCAGGTCCACTCTCGTGGTCTGCTCCTCTGA<br>AATGTATTAGTGGGTTGTGCCCCTAATCTATTGGTGTGATAATTATCTACGCCGTG<br>GATTGGGTTGGTTACTTGAAGACCTGCTTCTAACCGTCCCTTGGACAACTTTTGAC<br>AATTTTGACCTCAAATCAGGCA	ITS	580	MW081266.1
3	*Agaricales* spp.	**AACCCGGCCTTGCGGCGTGCTGAGTTCCTCAGTCCCAACCATTGTATACGAC<br>CAGAGGCTATAACACATCCGAAGATGCCACATTCCCCTGACCTTTATCCAAC<br>GGTCAAAACTGATGTTGACCCGTCAACTAGGAAGTACACCAATCAGAAGAAA<br>GGTTGATCCCCAGCTAACGCGACTGACTTCAAGCGTTTCCCTTTCAGCAATTT<br>CACGTACTGTTTAACTCTCTTTCCAAAGTTCTTTTCATCTTTCCCTCACGGTA<br>CTTGTTCGCTATCGGTCTCTCGCCAATATTTAGCTTTAGATGGAATTTACCAC<br>CCATTTAGAGCTGCATTCCCAAACAACTCGACTCTTCGAGAGCGTATCACAA<br>AGCCCTGGTAGTCCGTGTCAAAGACGGGATTCTCACCCTCTATGATGCTCCC<br>TTCCAGGAGACTTATACACGGTCCAGCGCGGATAACACTTCTCTAAATTACA<br>ACTCGGACAGCCAAAGACTGCCAGATTTTAAATTTGAGCTTTTCCCGCTTCAC<br>TCGCAGTTACTAGGGGAATCCTTGTT**	D1D2	550	MW557949.1
4	*Candolleomyces* spp.	TTGTAGCTGGCTCCTAGGAGCATTTGTGCACGCCCGTCATTCATATCATCTTTCCA<br>CCTGTGAACTATGTGTAGGCCTGGATCCCCCTCGCTTTGGCAACAAAGCGGATGC<br>AAGGATTGCTGTGTCGACAAGACCGGCTCTCTTTGAATTTCCAGGTTCTATGTTTT<br>TTACACACCCCATTTGAATGATGTAGAATGTTGTCAAATGGGCTTTTATGCCTATA<br>AAACACTATACAACTTTCAGCAACGGATCTCTTGGCTCTCGCATCGATGAAGAAC<br>GCAGCGAAATGCGATAAGTAATGTGAATTGCAGAATTCAGTGAATCATCGAATCT<br>TTGAACGCACCTTGCGCTCCTTGGTATTCCGAGGAGCATGCCTGTTTGAGTGTCAT<br>TAAATTCTCAACCTCACCAGTTTTGTAACGAGACAGGTGAAGGCTTGGATGTGGG<br>AGTTTTTGCAGGCTGCCTCAGTGCTGGTCTGCTCTCCTGAAATGCATTAGCGAGCT<br>CATACTGAGCTTCCGTCTATTGGTGTGATAATTATCTACGCCGTGGAATTGGACTC<br>ATGCTTGCTTCTAACCGTCCGCAAGGACAATCTACTTGACCAATTTGACCTCAAAT<br>CAGGTAGG	**ITS**	**620**	PV658776.1
5	*Schizophyllum commune*	**GTCAAGTCAAAAGTTAGCGCACAAGTCGCTAGTCTCGGTCAAGAGACGGTTG<br>GAAGCAGACTCCTATTGAAACTGACTAGGTCAGCCCCGAGATGGTCAACGAC<br>GTAGAAATTATCACATCGGAGACGCGATCCCGCAAGGGAAATCCGCTAATAC<br>ATTTAAGAGGAGCTGGCTCCGTTAGGCTCCAGCAGACCTCCACTTCCAAGCC<br>ACTCTCGAGACCGAAGTCAAAAGAGGGTTGATGGTATTTAATGACACTCAAA<br>CAGGCATGCCCCTCGGAATACCAAAGGGCGCAAGGTGCGTTCAAAGATTCGA<br>TGATTCACTGAATTCTGCAATTCACATTACTTATCGCATTTCGCTGCGTTCTT<br>CATCGATGCGAGAGCCAAGAGATCCGTTGTCGAAAGTTGTATTAACTTTTTA<br>GGGTCTGTCAAGACCATGATTACATTCGTTAACATACTTTAAGGTGTGAGGT<br>AGACGTAGTCAACCGCCGCCCGTGAAGGCTTTGGGACTACATAAGGTGCACA<br>GGATCAGAACAAGATGAACTTGTTTGATT**	ITS	550	MK353082.1

6	*Schizophyllum commune*	GTGCACCTTATGTAGTCCCAAAGCCTTCACGGGCGGCGGTTGACTACGTCTACCTC<br>ACACCTTAAAGTATGTTAACGAATGTGATCATGGTCTTGACAGACCCTAAAAAGT<br>TAATACAACTTTCGACAACGGATCTCTTGGCTCTCGCATCGATGAAGAACGCAGC<br>GAAATGCGATAAGTAATGTGAATTGCAGAATTCAGTGAATCATCGAATCTTTGAA<br>CGCACCTTGCGCCCTTTGGTATTCCGAGGGGCATGCCTGTTTGAGTGTCATTAAAT<br>ACCATCAACCCTCTTTTGACTTCGGTCTCGAGAGTGGCTTGGAAGTGGAGGTCTGC<br>TGGAGCCTAACGGAGCCAGCTCCTCTTAAATGTATTAGCGGATTTCCCTTGCGGGA<br>TCGCGTCTCCGATGTGATAATTTCTACGTCGTTGACCATCTCGGGGCTGACCTAGT<br>CAGTTTCAATAGGAGTCTGCTTCCAACCGTCTCTTGACCGAGACTAGCGACTTGTG<br>CGCTAACTTTTGACTTGACCTCAAATCAGGTAGGACTACCCGCTGAACT	ITS	550	MW577332.1
7	*Fomitopsis* spp.	GCATTGTCCCCAAGTAAAGGACGATTGGAAGCCGATCCCATTAACATGCTTCACA<br>GCAACGGCGTAGACAATTATCACACCGATAGCTGATCCACAAAGGTTCGAGCTAA<br>TGCATTCAAGAGGAGCCGATCACAGGTGTACCGGCAATAAACCTCCAAGTCCAAG<br>CCCTATTCACAAAAGTGAATAGAGTTGAGAATTCCATGACACTCAAACAGGCATG<br>CTCCTCGGAATACCAAGGAGCGCAAGGTGCGTTCAAAGATTCGATGATTCACTGA<br>ATTCTGCAATTCACATTACTTATCGCATTTCGCTGCGTTCTTCATCGATGCGAGAG<br>CCAAGAGATCCGTTGCTGAAAGTTGTATTTAAATGCGTTAGACGCAACAGTGACA<br>TTCTTTAAACTGAAGTAGTTTGTAATAAAACATAGGAAGGCCTACAACCAAAGCG<br>GACACAGAGCGCGCCCACTCCAGCCACAAACCGACCTACAGTGTGTGCACAGGTG<br>TTTGAGATGGATAATGATCAGGGCGTGCACATGCCGCTGACGGCCAGCAACAACC<br>CCGTTCAGA	ITS	550	MZ497289.1
8	*Geliporus exilisporus*	GCGAGTGAGCGGGAAAAGCTCAAATTTAAAATCTGGCAGTCTTTGGCTGTCCGAG<br>TTGTAGTCTGGAGAAGCGTTTTCCGCGCTGGACCGTGTACAAGTCTCCTGGAACG<br>GAGCGTCATAGAGGGTGAGAATCCCGTCTTTGACACGGACTACCAGTGCTTTGTG<br>ATGCGCTCTCAAAGAGTCGAGTTGTTTGGGAATGCAGCTCAAAATGGGTGGTAAA<br>CTCCATCTAAAGCTAAATATTGGCGAGAGACCGATAGCGAACAAGTACCGTGAGG<br>GAAAGATGAAAAGCACTTTGGAAAGAGAGTTAAACAGTACGTGAAATTGCTGAA<br>AGGGAAACGCTTGAAGTCAGTCGCGTTTGCTAGAACTCAGCCTGGCCTCGGCTTG<br>GTGCATTTTCTAGTGAACGGGCCAGCATCACTTTTGGTTGCGGGATAAAGGTCGC<br>AGGAATGTGGCACCTTCGGGTGTGTTATAGCCTGTGATTGTATACCGTGGCTGGG<br>AGTGAGGAACTCAGCACGCTCACGCTGTGCTTAGGATGCTGGCGTAATGGCTTTA<br>A	D1D2	550	LC379153.1
9	***Odontoefibula orientalis***	GTCTCAATCTGAGGCATGTGCACGCCCTACTCATCCACTCTCAACCTCTGTGCACT<br>TACTGTAGGTCGGTGAAAGGTTCGCTCTGCTAGCTGGAAGCCTTCCTATGTTTCAC<br>TACAAACGCTTCAGTTTTAGAATGTAACCTTGCGTATAACGCATCTATATACAACT<br>TTCAGCAACGGATCTCTTGGCTCTCGCATCGATGAAGAACGCAGCGAAATGCGAT<br>AAGTAATGTGAATTGCATAATTCAGTGAATCATCGAATCTTTGAACGCACCTTGCG<br>CTCCCTGGTATTCCGGGGAGCACGCCTGTTTGAGTGTCATGGTATTCTCAACCTTC<br>ATAACTTTTGTTATCGAAGGCTTGGATTTGGAGGTTGTGCTGGCTCTCGTTAATCG<br>GCTCCTCTTAAATGCATTATCGTGAACGTTTGCCGATCGCTATCAATGTGATAATT<br>ATCTGCGCTGTTAATGTTGAAGCATCATTAGTGTCGCGCTTCTAACCGTCCTTCAC<br>AGGACAACTTACTTTGACATCTGACCTCAAATCAGGCGGGACTACCCGAAGGAAC<br>TTAAGCATA	ITS	567	NR_175609.1
10	*Irpex laceratus*	GATTTGAGCTCAGATTGTCATGAATTGTCTCGGTAAGAGACGACTATAAGCATGA<br>ACTAATAAATACTTCAACACCACAGCGCAGATAATTATCACACTGAAGGCGATCC<br>GTAAGATTCACGCTAATACATTTCAGAGGAGTCGACTAGAAGCCGACACAACCTC<br>CAAGTCCAAGCCCACTAAACTTCATTACAAAATTTAGGGGTTGAGAATTCCATGA<br>GACTCAAACAGGCATACTCCTCGGAATACCAAGGAGTGCAAGGTGCGTTCAAAGA<br>TTCGATGATTCACTGAATTCTGCAATTCACATTACTTATCGCATTTCGCTGCGTTCT<br>TCATCGATGCGAGAGCCAAGAGATCCGTTGCTGAAAGTTGTATATAATTGTGTTAT<br>ACACAGTAAACATTCTATAACTGAAGCGTTTGTAGTAAACATAAGAAAGGCTTAT<br>TACCGACTATTAAATAGCTGGCTTACACCGTTTCTTACATAAAGTGCACAGAGGTT<br>GAGAGTGGATGAGCCAGGCGTGCACATACCTCGTTAAAGGCCAGCTACAACCCGT<br>TCAAAACTCGATAATG	ITS	570	KT844687.1
11	*Volvariella volvacea*	GGTCAGAGACCCGGCCTTTCGGCGCGCTGAGTTCCTCAGTCCCAACCGCCGCATA<br>CGACCAGAGGCTATAACACACCCAGAGGTGCCACATTCCTCAGGCCTTTTTCCGG<br>CGGTCGAAACTGATGCTGACCCGTCGACCGGGAAGTGCGCCCCGCAGTTGCAAGG<br>TTGATCCCCGGCCGACGCGACTGACTTCAAGCGTTTCCCTTTCAACAATTTCACGT<br>ACTGTTTAACTCTCTTTCCAAAGTGCTTTTCATCTTTCCCTCACGGTACTTGTTCGC<br>TATCGGTCTCTCGCCAATATTTAGCTTTAGATGGAGTTTACCACCCATTTTGAGCT<br>GCATTCCCAAACAACTCGACTCTTCGAGAGCGCACCACAAAAGCCCTGGGAGTCC<br>GTGCCAAAGACGGGATTCTCACCCTCTGTGACGCTCCGTTCCAGGAGACTTGTGC<br>ACGGTCCAGCGCGGGTAGCACTTCTCTAGATTACAACTCGGACGGCCAAGGACCG<br>CCAGATTTCAAATTTGAGCTCTTCCCGCTTCACTCGCAGTTACTAGGGGAA	D1D2	550	MN738572.1
12	*Candollemyces*	GCCCGTCATTCATATCATCTTTCCACCTGTGAACTATGTGTAGGCCTGGATACCCC<br>TCGCTTTGGCAACAAAGCGGATGCAAGGATTGCTGCGTCGACAAGGCCGGCTCTC<br>TTTGAACTTCCAGGTTCTATGTCTTTACACACCCCATTTGAATGATGTAGAATGT	ITS	640	PV155134.1

	rubrobrunneus	AGTCAATGGGCTTTCATGCCTATAAAACACTATACAACTTTCAGCAACGGATCTCTTGGCTCTCGCATCGATGAAGAACGCAGCGAAATGCGATAAGTAATGTGAATTGCAGAATTCAGTGAATCATCGAATCTTTGAACGCACCTTGCGCTCCTTGGTATTCCGAGGAGCATGCCTGTTTGAGTGTCATTAAATTCTCAACCTCACCAGTTTTGTAACGAGACCGGTGAAGGCTTGGATGTGGGGGTTTTGCAGGCTGCCTCAGTGCTGGTCTGCTCCCCTGAAATGCATTAGCGAGCTCATACTGAGCTTCCGTCTATTGGTGTGATAATTATCTACGCCGTGGATTGGACTCATGCTTGCTTCTAACCGTCCGCAAGGACAATTGACTTGACCAATTTGACCTCAAATCAGGTAGGACTACC			
13	*Schizophyllum commune*	CTGGCGGTCTCCGGCCGTCCGAGTTGTAATTTAGAGAAGCGTTATCCGTGCTGGACCGTGTATAAGTCTCCTGGAATGGAGCGTCATAGAGGGTGAGAATCCCGTCTTTGACACGGACTACCAGTGCATTGTGATGCGCTCTCGACGAGTCGAGTTGTTTGGGAATGCAGCTCAAAATGGGTGGTAAATGCCATCTAAAGCTAAATATTGGCGAGAGACCGATAGCGAACAAGTACCGTGAGGGAAAGATGAAAAGAACTTTGGAAAGAGAGTTAAACAGTACGTGAAATTGTCGAAAGGGAAACGGTTGCAGTCAGTCGCGTCCGCCGGGGATCAACCTTGCTTTTGCTTGGCGTATTTCCCGGTGGATGGGTCAGCATCAGTTTTGACCGCAGTTGAAAGGCTGGAGGAATGTGGCACCTTCGGGTGTGTTATAGCCTCCTGTCATATGCTGCGGTTGGGACTGAGGAACTCAGCACGCCGCAAGGCCGGGATTCGTCCACGTTCGTGCTTAGGATGCTGGCATAATGT	D1D2	570	PX21101 3.1
14	*Deconica coprophila*	TCGAGAGTATGTGCACGCTCATCATCTTTATATTTCCACCTGTGCACCTTTTGTAGACCCAGGCGACTTGAGCAATCAAGTCAATTGAGTCTATGTCTTATCATATACCCCAAAAGCATGTCTTTGAATGTATCAATGGGCTTTGATGCCTATAAACATTATACAACTTTCAGCAACGGATCTCTTGGCTCTCGCATCGATGAAGAACGCAGCGAAATGCGATAAGTAATGTGAATTGCAGAATTCAGTGAATCATCGAATCTTTGAACGCACCTTGCGCTCCTTGGTATTCCGAGGAGCATGCCTGTTTGAGTGTCATTAAATTCTCAACCTCTCTAGTTTTTTGACTAGTAGATGGCTTGGATGTGGGGGTTTTGTGCAGGCTTTCACAAGTCAGCTCCCCTGAAATGCATTAGCCGGTACTTTCTGACCGTCTATTGGTGTGATAATTATCTACGCCGTGGACTATTGGATAAGTGTACTGCTTCTAATCGTCTTTTACTAGACAATTACATGACATTTTGACCTCAAATCAGGTAGG	ITS	541	OP237142.1

Supplementary File S1. STROBE Statement—Checklist of items that should be included in reports of ***cohort studies***

	Item No	Recommendation	Page (line)
**Title and abstract**	1	(*a*) Indicate the study's design with a commonly used term in the title or the abstract	1 (17)
		(*b*) Provide in the abstract an informative and balanced summary of what was done and what was found	1 (22)
**Introduction**			
Background/rationale	2	Explain the scientific background and rationale for the investigation being reported	2 (49)
Objectives	3	State specific objectives, including any prespecified hypotheses	3 (93)
**Methods**			
Study design	4	Present key elements of study design early in the paper	9 (324)
Setting	5	Describe the setting, locations, and relevant dates, including periods of recruitment, exposure, follow-up, and data collection	9 (325)
Participants	6	(*a*) Give the eligibility criteria, and the sources and methods of selection of participants. Describe methods of follow-up	9 (328)
		(*b*) For matched studies, give matching criteria and number of exposed and unexposed	NA
Variables	7	Clearly define all outcomes, exposures, predictors, potential confounders, and effect modifiers. Give diagnostic criteria, if applicable	9 (335)
Data sources/ measurement	8*	For each variable of interest, give sources of data and details of methods of assessment (measurement). Describe comparability of assessment methods if there is more than one group	10 (358)

Bias	9	Describe any efforts to address potential sources of bias	9 (328)
Study size	10	Explain how the study size was arrived at	9 (328)
Quantitative variables	11	Explain how quantitative variables were handled in the analyses. If applicable, describe which groupings were chosen and why	10 (386)
Statistical methods	12	(*a*) Describe all statistical methods, including those used to control for confounding	NA
		(*b*) Describe any methods used to examine subgroups and interactions	10 (386)
		(*c*) Explain how missing data were addressed	NA
		(*d*) If applicable, explain how loss to follow-up was addressed	NA
		(*e*) Describe any sensitivity analyses	NA
**Results**			
Participants	13*	(a) Report numbers of individuals at each stage of study—eg numbers potentially eligible, examined for eligibility, confirmed eligible, included in the study, completing follow-up, and analysed	3 (107)
		(b) Give reasons for non-participation at each stage	NA
		(c) Consider use of a flow diagram	NA
Descriptive data	14*	(a) Give characteristics of study participants (eg demographic, clinical, social) and information on exposures and potential confounders	3 (107)
		(b) Indicate number of participants with missing data for each variable of interest	NA
		(c) Summarise follow-up time (e.g., average and total amount)	NA
Outcome data	15*	Report numbers of outcome events or summary measures over time	13 (489)
Main results	16	(*a*) Give unadjusted estimates and, if applicable, confounder-adjusted estimates and their precision (e.g., 95% confidence interval). Make clear which confounders were adjusted for and why they were included	NA
		(*b*) Report category boundaries when continuous variables were categorized	3 (107)
		(*c*) If relevant, consider translating estimates of relative risk into absolute risk for a meaningful time period	NA
Other analyses	17	Report other analyses done—eg analyses of subgroups and interactions, and sensitivity analyses	NA
**Discussion**			
Key results	18	Summarise key results with reference to study objectives	6 (175)
Limitations	19	Discuss limitations of the study, taking into account sources of potential bias or imprecision. Discuss both direction and magnitude of any potential bias	8 (295)
Interpretation	20	Give a cautious overall interpretation of results considering objectives, limitations, multiplicity of analyses, results from similar studies, and other relevant evidence	8 (295)
Generalisability	21	Discuss the generalisability (external validity) of the study results	9 (305)
**Other information**			
Funding	22	Give the source of funding and the role of the funders for the present study and, if applicable, for the original study on which the present article is based	11 (412)

*Give information separately for exposed and unexposed groups.

Note: An Explanation and Elaboration article discusses each checklist item and gives methodological background and published examples of transparent reporting. The STROBE checklist is best used in conjunction with this article (freely available on the Web sites of PLoS Medicine at http://www.plosmedicine.org/, Annals of Internal Medicine at http://www.annals.org/, and Epidemiology at http://www.epidem.com/). Information on the STROBE Initiative is available at http://www.strobe-statement.org.

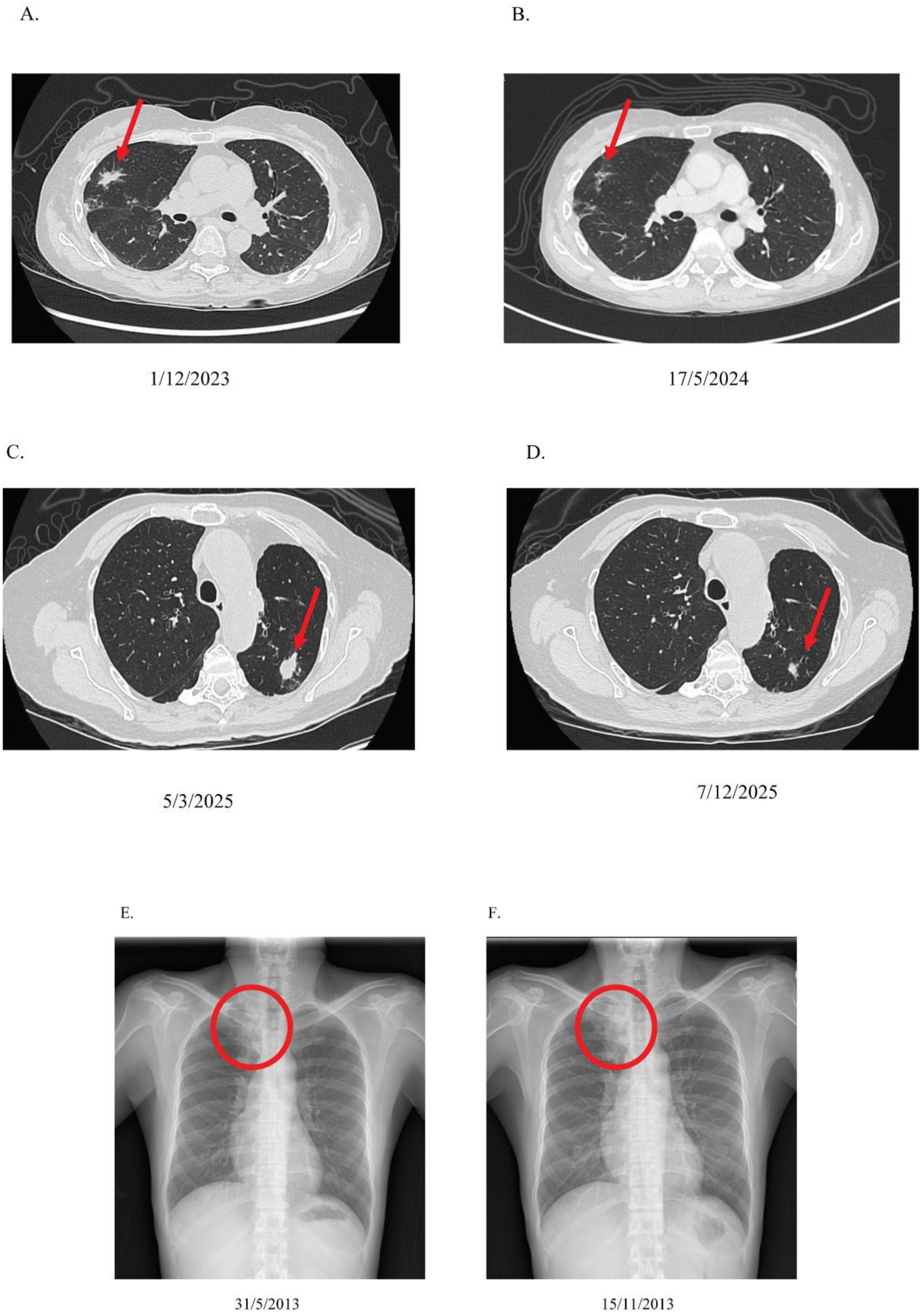

Supplementary Figure S1. Contrast-enhanced chest CT images of patient no. 6 before treatment (A) and after treatment (B) demonstrate radiologic improvement. Contrast-enhanced chest CT images of patient no. 13 before treatment (C) and after treatment (D) also demonstrate radiologic improvement. In contrast, chest radiographs of patient no. 5 obtained prior to treatment (E) and following treatment (F) demonstrate radiologic progression.
